# Component Rhinoplasty

**Published:** 2014-01

**Authors:** Muhammad Humayun Mohmand, Muhammad Ahmad

**Affiliations:** Cosmetic Plastic Surgeon, La Chirurgie, Islamabad Cosmetic Surgery Centre, Islamabad, Pakistan

**Keywords:** Component, Rhinoplasty

## Abstract

**BACKGROUND:**

According to statistics of American Society of Plastic Surgeons, cosmetic rhinoplasty was the second most frequently performed cosmetic surgery. This study shares the experiences with component rhinoplasty.

**METHODS:**

From 2004 to 2010, all patients underwent aesthetic nasal surgery were enrolled. The patients requiring only correction of septal deviation and those presenting with cleft lip nasal deformity were excluded. All procedures were performed under general anaesthesia with open technique using transcolumellar and infra-cartilageous incisions. The transculomelalr incision was closed with 6-0 polypropylene and infra-cartilagenous incisions by 5-0 absorbable sutures. Non-absorbable sutures were removed on the fifth postoperative day. The septum was fixed by ‘quilting sutures’. Both nostrils were packed with antibiotic ointment containing paraffin gauzes which were removed after 24-48 hours. External nasal splint was applied to be removed on the fifth postoperative day.

**RESULTS:**

A total of 191 patients were enrolled (male:female ratio=1:1.47). The mean age of female patients was 25.3 years and 29.4 years in males. Among 50.8% of patients, the cause of deformity was not known. Only 21.5% patients had a positive history of trauma. Majority of patients (90.6%) underwent septoplasty. Twenty percent of surgeries were secondary. Spreader grafts were used in 85% of patients. In 11% of patients, conchal grafts were used. For none of patients, the inferior turbinectomy was performed. No case of costal cartilage graft or silicone implant was used. Only 5.6% of patients had redo-surgeries. No abnormal scarring was noted during follow-up.

**CONCLUSIONS:**

Dorsal hump reduction can be recommended with accuracy and safety without compromising the nasal airway.

## INTRODUCTION

Cosmetic rhinoplasty is the surgery to reshape the nose. It is perhaps one of the most fascinating of all aesthetic procedures. According to the statistics of American Society of Plastic Surgeons, cosmetic rhinoplasty was the second most frequently performed cosmetic surgical procedure.^1^ The incidence of aesthetic rhinoplasty is increasing in the Asians.^2^ The main objective of the surgery for Asians is to modify the nose such that the facial results compare favourably with the current aesthetic ideals for white people.^2^ The difference between a good and a poor result can be 1 tot 2 mm.^3^

The external approach provides a wide undistorted exposure to the bony and cartilaginous framework of the nose, allowing the accurate evaluation and precise surgical control.^4^ An aesthetically pleasing good dorsal nasal profile is a sinequanon for a successful rhinoplasty result. 

The composite reduction of the nasal dorsum refers to reducing all or most of the components of the nasal dorsum ‘en block’ (septum, upper lateral cartilages, perpendicular plate of the ethmoid, nasal bones, and vestibular mucosa) preferably with a straight cutting osteotome.^5^^-^^8^ This technique leaves a little room for errors, is more difficult to control, and commits the surgeon to discard the upper lateral cartilage with the initial resection. If the resection is more than 2 mm, the component reduction seems the most logical option.

The component reduction of the nasal dorsum refers to the reducing each component of the nasal dorsum individually, i.e., septum, upper lateral cartilages, bone and mucosa.^5^ The advantages of the component reduction are that it maximizes the accuracy and control of resection while allowing selective preservation of any of the four elements of the dorsum relative to the others. This maintains the integrity of the dorsal vestibular mucosa for possible later use as a spreader graft and preserves the dorsal upper lateral cartilages. The purpose of the present study was to share our experiences with the component rhinoplasty technique and to assess the aesthetic results.

## MATERIALS AND METHODS

The study was conducted in a private setup from 2004 to 2010. All those patients who underwent aesthetic nasal surgery were included in the study. The patients requiring only the correction of septal deviation were excluded. Similarly, patients presenting with cleft lip nasal deformity were also excluded from the study.

All the patients were evaluated with detailed history and a thorough physical examination and also preoperative photographs were provided. All the procedures were performed under general anaesthesia with open technique using the transcolumellar and infra-cartilageous incisions.

**Fig. 1:**
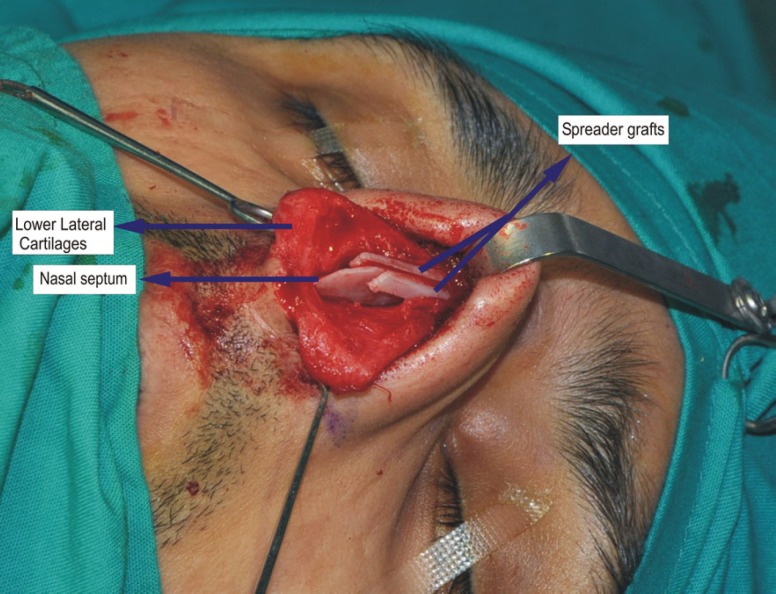
Transcolumellar and infracartilagenous incision

In all the patients, 1% lidocaine with 1:100,000 epinephrine was used to infiltrate the area. A stair-step transcolumellar incision was used which was continued on either side as the infra-cartilagenous incisions. The soft tissue envelope was sharply elevated in a submucoperichondrial plane up to the bony pyramid. The periosteum was sharply incised and elevated in the subperiosteal plane up to the radix area. The upper lateral cartilages were sharply separated from the junction with the septum without damaging the underlying mucosa. Once these cartilages were separated completely, the anatomic structure became in three parts, i.e., septum in the middle and the upper lateral cartilages on either side. Here the resection of the dorsal septum was performed under direct vision (using no.15 blade). After cartilaginous septal resection, the bony hump was resected using the rasp (if less than 2 mm) or guarded osteotome (if more than 2 mm). At this point, the ostetomies were performed using right and left guarded, angled ostetomes intranasally. After the osteotomies, the resection of the upper lateral cartilages was performed. In cases, the spreader graft was needed, it was harvested from the nasal septum leaving 2 cm strut superiorly and caudally. The spreader grafts were fixed in the tunnel between the septum and upper lateral cartilages (Figure 1). At all the times, the assessment of the nasal shape was performed. 

**Fig. 2:**
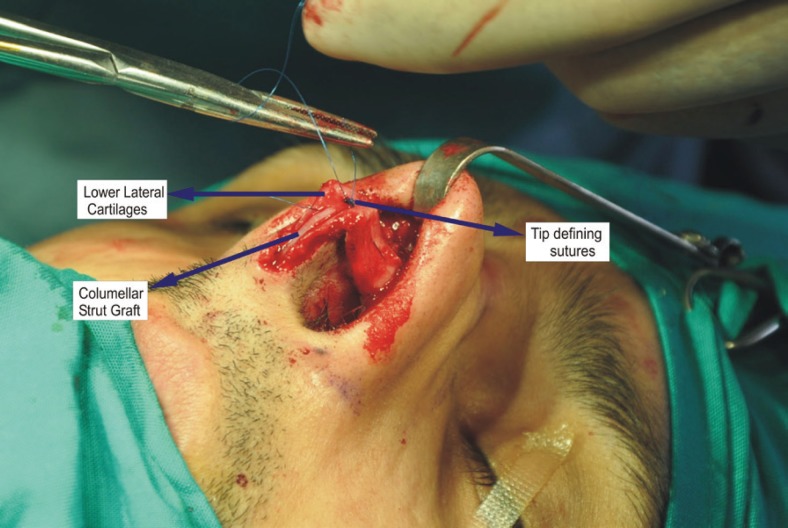
Nasal dorsum anatomy

The lower lateral cartilages were fixed using non-absorbable 6-0 prolypropylene. The nasal tip profile was enhanced by using the columellar strut graft with tip grafts and tip contouring with sutures (Figure 2). The transculomelalr incision was closed with 6-0 polypropylene and the infra-cartilagenous incisions were closed using 5-0 absorbable sutures. The non-absorbable sutures were removed on the fifth postoperative day. The septum was fixed by ‘quilting sutures’. Both nostrils were packed with antibiotic ointment containing paraffin gauzes which were removed after 24-48 hours. External nasal splint was applied to be removed on the fifth postoperative day.

## RESULTS

A total of 191 patients were included in the study with a male to female ration of 1:1.47. The mean age in female patients was 25.3 years (range 17-41 years) a s compared to 29.4 years (range, 16-40 years) in male patients. In most of the patients (50.8%), the cause of the deformity was not known (Table 1). Only 21.5% patients had a positive history of trauma. Majority of the patients (90.6%) underwent septoplasty as well. Twenty percent of the surgeries were secondary. More importantly spreader grafts were used in 85% of the patients. In 11% of the patients, conchal grafts were used. In no patient, the inferior turbinectomy was performed. No case of costal cartilage graft or silicone implant was used. Only 5.6% of the patients had redo surgeries. No abnormal scarring was noted during the follow-up of the patients (Table 2).

**Table 1:** Aetiologies of component rhinoplasty

**Cause**	**No. **	**Percent**
Congenital	13	6.8
Trauma	41	21.5
Previous surgery	39	20.4
Infection	1	0.5
Unknown	97	50.8

**Table 2:** Operative Options in component rhinoplasty

**Option **	**No. **	**Percentage**
Septoplasty	173	90.6
Speader grafts	163	85.3
Conchal grafts	21	11.0
Alar rim grafts	6	3.1

**Fig. 3:**
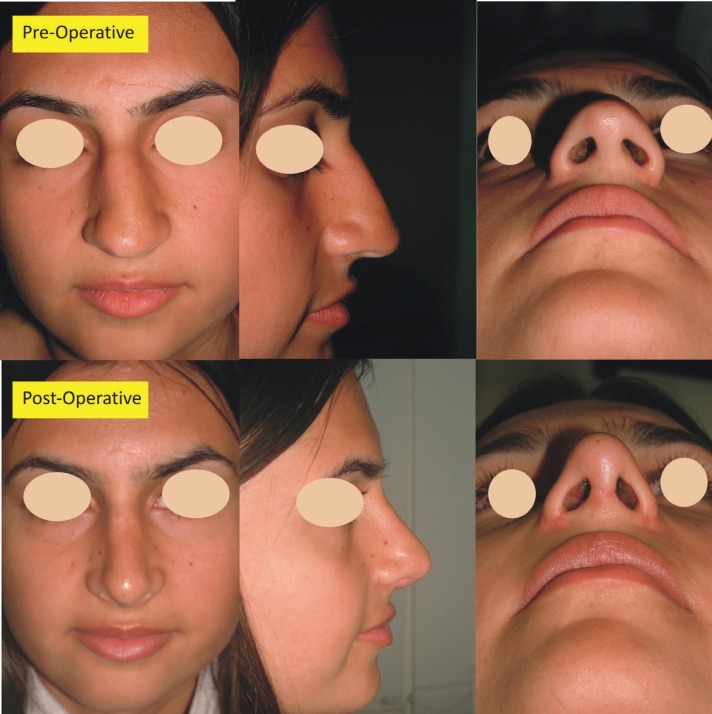
Patient with dorsal nasal hump and deviated septum

**Case 1: **A 21 years old female presented with depressed nasal dorsum and deviated septum (Figure 3). She underwent component reduction of nasal dorsum and was satisfied with the outcome.

**Fig. 4:**
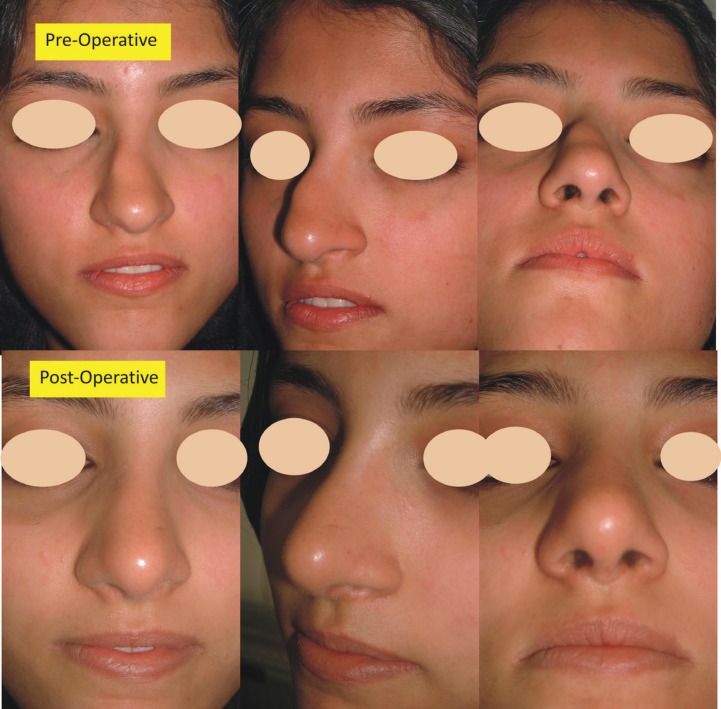
A young female with dorsal hump and dorsal nasal septal deviation

**Case 2: **A 23 years old female presented for the correction of dorsal nasal hump and septal deviation (Figure 4). She underwent component reduction rhinoplasty and had satisfactory result.

**Fig. 5:**
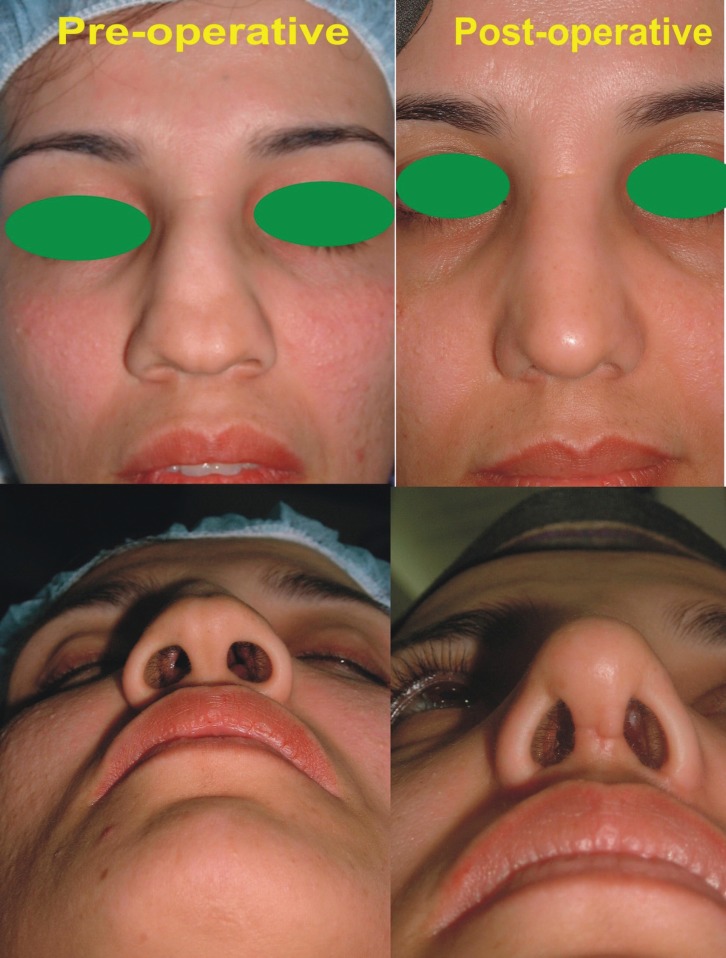
A young female with depressed nasal dorsum

**Case 3: **A 34years female had a post-traumatic depressed nasal dorsum and underwent the reduction rhinoplasty (Figure 5). She had satisfactory post-operative result.

## DISCUSSION

The basic aim of the rhinoplasty is the straight, smooth dorsum.^3^ The results of rhinoplasty are dependent on the anatomical knowledge of the surgeon, the severeness of deformity, preoperative plan and operative execution to obtain the preset goals. There are a number of ways to achieve an equally good result, with no right or wrong answer fort rhinoplasty^ 9^.

The component reduction of nasal dorsum refers to reducing each component of nasal dorsum individually-septum, upper lateral cartilages, bones and mucosa.^5^ Rochrich *et al. *suggest a five step approach to avoid the risk of asymmetry and over or under reduction; i) Separation of the upper lateral cartilages from the septum, ii) Gradual reduction of the septum, iii) Lowering of the bony dorsum, iv) Assessing the reduction by digital palpation, and v) Final modifications such as smoothening the edges, suturing triangular cartilages back to the septum, osteotomies or spreader grafts if needed.^8^

This approach provided the degrees of different resection levels for each component to achieve the best result. The most important point remains the separation of dorsal upper lateral cartilages from the dorsal septum, keeping the vestibular mucosa intact. The mobilization is stopped at the bony-cartilaginous junction.^10^ The dorsal septum is gradually cut followed by bony hump reduction. This allows the preservation of the upper lateral cartilages preventing stenosis and subsequent nasal airway obstruction.^11^^,^^12^

The inverted V-deformity is often the result of excessive removal of transverse portion of the upper lateral cartilages during the dorsal septal resection.^7^ The spreader grafts harvested from the septum can also be sutured on one or both sides of the dorsal septum to achieve the natural aesthetic lines. An all the cases, we used internal low-to-low or low-to-high osteotomies, depending on the individual case. We did not use the transcutaneous osteotomies due to the fact of excessive scar marks/pigmentation due to climate and skin types. The typical subcontinent nose lacks projection. Some noses may even need augmentation at one place and reduction at the other.^13^

Hence the surgeon must be well-versed with the techniques of cartilage grafting. We also performed nasal tip reshaping by using the sutures techniques to alter the shape of the lower lateral cartilages.^14^ So dorsal hump reduction can be recommended with accuracy and safety without compromising the nasal airway.

## Footenote

Please cite this paper as:

Mohmand MH, Ahmad M. Component rhinoplasty. World J Plast Surg 2014;3(1):18-23.
